# Molecular structure data and modelling roadmap for optimized oxidized graphene quantum dot and epoxy interface and mechanical properties

**DOI:** 10.1016/j.dib.2024.111059

**Published:** 2024-10-22

**Authors:** Prathamesh Deshpande, Robert Chan-Jobe, Ozgur Keles

**Affiliations:** San Jose State University, 1 Washington Sq., San Jose, CA 95192, United States

**Keywords:** Molecular dynamics, Graphene oxide quantum dots, Epoxy nanocomposite, Molecular interactions, Mechanical response

## Abstract

Hybrid epoxy composites are highly considered for low-density applications due to the excellent specific strength and specific stiffness. Enhancements made to the epoxy matrix by addition of nanofillers like carbon nanotubes (CNTs) and graphene (GNPs) have been studied in detail over the course of few decades. These enhancements not only help elevate the material properties of the matrix but also activate different failure mitigating mechanisms in the composite. Although highly beneficial, there are few shortcomings due to the challenging fabrication process of integrating such. Common problems like filler agglomeration, formation of voids, wrinkling and more can result in poor load transfer within the composite. Graphene quantum dots (GQDs), on the other hand are the smallest carbon-based filler which are known to promote more intimate contact with the matrix. Their small size enables simultaneous enhancement of stiffness, strength and toughness. In addition, functionalization of these materials enables other supramolecular interactions like hydrogen bonding which improve the interfacial interaction with the epoxy. This study provides a molecular dynamics (MD) workflow to model a single functionalized GQD embedded in an epoxy matrix and the effective mechanical response of the nanocomposite. Ten chemistries were developed with different oxygen-based functional groups which capture the effect of GQD on the mechanical properties of the nanocomposite. Uniaxial strain simulations revealed that a maximum strength gain of 56 % and stiffness gain of 18 % was computed by the oxidized GQD-epoxy nanocomposite.

Specifications Table

**Table:** 

Subject	Physical and Theoretical Chemistry, Computational Mechanics, Computational Mechanics
Specific subject area	Molecular Dynamics nano-structure development and mechanical property prediction of bonded and non-bonded oxidized GQD-epoxy nanocomposites
Data format	Raw: Molecular structure files, LAMMPS input scripts and LAMMPS auxiliary files Analysed: LAMMPS output structure files and stress-strain plots
Type of data	PDB and MOL raw molecular structures, CSV files with output data, LAMMPS input scripts.
Data collection	Raw PDB and MOL structures were developed using AVOGADRO. Ten GQD chemistries were developed. LUNAR toolkit was used to transform the raw inputs into usable files for performing LAMMPS simulation and predicting the mechanical properties. Purdue Anvil was used to perform the MD simulations through NSF ACCESS. Some simulations were performed on College of Engineering HPC at San Jose State University.
Data source location	San Jose State University, San Jose, CA 95,192
Data accessibility	Repository name: Mendeley Data Data identification number: 10.17632/nvrkj7cygx.2 Direct URL to data: https://data.mendeley.com/datasets/nvrkj7cygx/2

## 1 Value of the Data

• The comprehensive molecular dynamics data provides effects of oxidation of GQDs on the GQD-epoxy nanocomposites. The mechanical response and nano-structural configuration reveals the strengthening mechanisms such as the effect of supramolecular interactions.
• The impact of non-bonded and bonded interactions on the mechanical properties are critical for designing hybrid polymer composites that maximize the mechanical performance of the material. This work encapsulates a wide range of GQD chemistries which be used for intelligent material design.
• Material scientists and manufacturers invested in hybrid polymer composites can leverage this work to understand the molecular behaviour of the material. Also, other computational researchers benefit from the new modelling methodology which includes implementation of the LUNAR toolkit.
• The data helps simplify a complex structure yet capture the critical molecular description and provides pathways to more complex modelling methods and analyses.

## 2 Background

Epoxy composites have gained excessive usability in various applications which includes adhesives, electronics, and mechanical structures. The low-density, high strength and high stiffness make them a versatile and low maintenance material. However, the epoxy matrix inherently is brittle and causes poor load transfer in the composite. Multiple studies have shown that epoxy matrix properties can be enhanced by addition of second phase materials like rubber, impact modifying thermoplastics, natural and carbon fibres, and nanofillers like carbon nanotubes, graphene platelets and quantum dots [1].

Quantum dots (QDs) are the smallest available nanofiller with sizes smaller than 20 nm [[2], [3], [4], [5], [6]]. Their size enables them to create greater contact with the matrix and help solve dispersion related problems. Graphene quantum dots (GQDs), both pristine and functionalized, have been studied to show increased mechanical properties in epoxies [2,4,5,7]. The property enhancements in GQD-epoxy composites have been attributed to higher dispersion of GQDs in epoxy and the resulting boost to the interfacial intermolecular interactions. To completely understand the mechanisms behind the improved material properties, computational tools like molecular dynamics (MD) can be used [[8], [9], [10], [11], [12], [13], [14]]. MD uses high-performance computing to reveal material behaviour at atom-scale.

In this study, we provide a comprehensive dataset of molecular structures through a validated modelling workflow for developing functionalized GQD-epoxy nanocomposite with oxidation effects. The GQDs are oxidized by attaching different oxygen-based functional groups which include the hydroxyl (-OH), carboxylic acid (-COOH), and epoxide (-(CH—O-CH_2_)). The different nanocomposites are evaluated for their mass densities and mechanical properties using a set of uniaxial tension simulations. The output structures and other output files can be further studied to reveal the evolution of the nano-structure. This detailed study will help guide the future experimental investigations by revealing the effects of oxidation densities, distribution and functionalities (Table 1).

**Table 1:** Directory data description for all the material systems.

Directory name	Simulation Details
input_scripts	LAMMPS simulation scripts for all the workflow steps
molecular_structure_files	Molecular structures for raw molecules, mixture models and all the subsequent simulation input and output files
	
reaction_files	REACTER molecule templates (pre- and post-reaction) and reaction map
reaxff_files	ReaxFF parameter set
stress-strain_csvfiles	Stress-strain output data for all the replicates in the three principal directions

## 3 Data Description

**Fig. 1:**
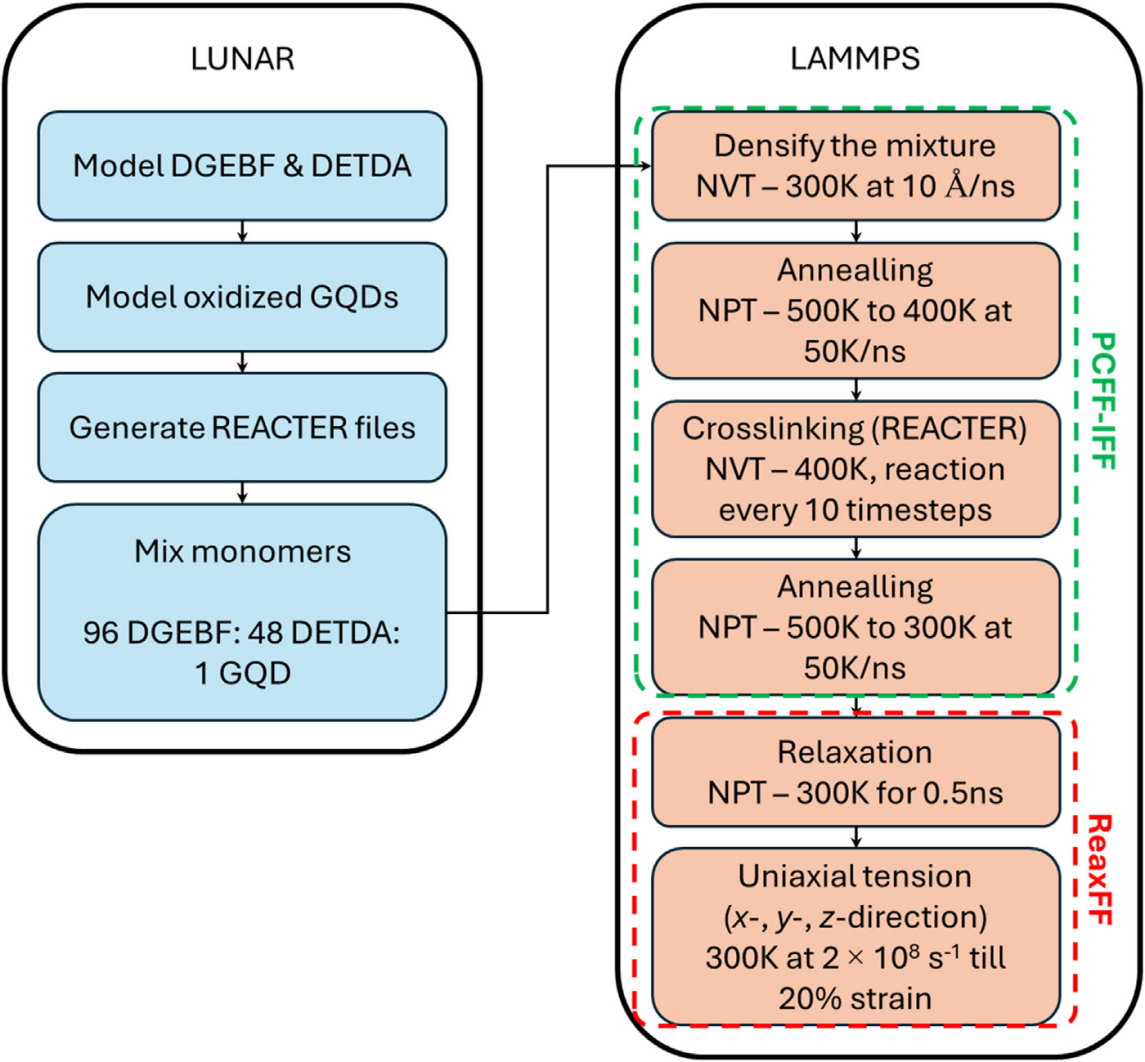
Molecular Modelling workflow.

The data is distributed in ten separate directories for the ten modelled material systems. The directory names are - e4OH-GQD, e8OH-GQD, s4OH-GQD, s8OH-GQD, s20OH-GQD, m8OH-GQD, s4O-GQD, e4COOH-GQD, m4O-4COOH-GQD, and m6O-6OH-GQD. Each directory contains five sub-directories which include the necessary input files for a all the LAMMPS simulations as shown in Fig. 1. It is important to note that the repository only contains files relevant for running LAMMPS simulations. The pre-processed files obtained from LUNAR are directly supplied since an example repository is already made available by the LUNAR developers [15]. This repository can be used to learn the utility and capability of LUNAR. The details of all the workflow simulations are listed in the Methods section. All the sub-directories contain the essential files for five replicates that are required to perform MD simulations using the LAMMPS software. The file details for all the different filetypes are listed in Table 2.

**Table 2:** MD simulation files including data description for all the contents.

Filetypes	File description
*.data or *.reaxdata	LAMMPS structure
in.*	LAMMPS input script
*.pdb and *.mol	Raw molecular input files
*.lmpmol	reaction template
*.txt	reaction map
*_rff.txt	ReaxFF atom assignments
*.csv	stress-strain data
*.reax	ReaxFF forcefield parameter file

The output (*.data) from the “input_scripts/in.sim1” simulation is to be used to run the next simulation (input_scripts/in.sim2) and so on. The output file that is essential for a continuous workflow is the “*.data” or the LAMMPS structure file. The other output files can be used for further analysis. File which is not included in the data repository is the “dump.*” or LAMMPS trajectory file. These files are usually large in size and mostly used for visualizations. When any of the six simulations are performed, the trajectory files will automatically get generated. Another file which is not included is the “rst.*” or LAMMPS simulation restart files. These are binary files which are automatically generated during a simulation run. They can be used to restart any simulation from an error state to the normal end of that simulation. Since these files are useful only for a failed simulation and they are automatically generated during the simulation, they are excluded from the dataset. Also, the log files from all the simulations are not included in the directories since they are dependent on simulation settings and are auto generated after each run. The final log files or csv files from the tension simulations are included for quick analysis.

## 4 Experimental Design, Materials and Methods

The modelled epoxy matrix in this study is the diglycidyl ether bisphenol F or DGEBF. The commercial product is known as EPON 862. The modelled curing agent is the diethyl toluene diamine or DETDA which is sold commercially as Epikure W. The modelled GQD is of zigzag structure and ∼2 nm diameter. A pristine GQD was functionalized using different oxidation groups. Total ten unique functionalized GQDs were modelled as shown in Fig. 1. The molecular models were generated using the LUNAR toolkit [15]. The MD simulations were performed on Anvil [16], a HPC at Purdue University, through NSF ACCESS [17]. A stepwise modelling workflow using LUNAR and LAMMPS is shown in Fig. 1. The LUNAR modeling details have been discussed elsewhere [15]. The detailed LAMMPS modelling is explained as follows-

1. The two epoxy monomers were mixed in a simulation box with a mixing ratio of 2(DGEBF):1(DETDA) using the LUNAR toolkit [15]. PCFF-IFF forcefield was used to define the interatomic interactions within the modelled molecules [12,18]. The selected mixing ratio was selected to maximize the chemical crosslinking between the molecules during the virtual cure [9,19,20]. The mixture was multiplied to generate a larger model with the total atom count of ∼5600 atoms. A single functionalized GQD was inserted at a random location in the simulation box by avoiding overlap with the epoxy molecules using the “cell_builder” tool within the LUNAR toolkit. Five replicates were generated for each of the ten GQD structures, bringing the total model count to 50. Each GQD replicate had random distribution of functional groups making them distinct structures with identical chemistries. The chemical structures of the modelled monomers are shown in Fig. 2. The GQD structure's naming convention includes letters “e”, “s”, or “m” in the first position which represents the location of attached functional groups; “e” for edge, “s” for surface, and “m” for mixed location or both edge and surface. The followed set of numbers and letters designates the total number of functional groups and the type of functional groups. For example, ***s***20OH-GQD includes 20 OH or hydroxyl groups on the surface or the basal plane of the GQD. Also, in case of ***m***4O-4COOH-GQD and ***m***6O-6OH-GQD there are two different functional groups attached to GQD in mixed locations.

**Fig. 2:**
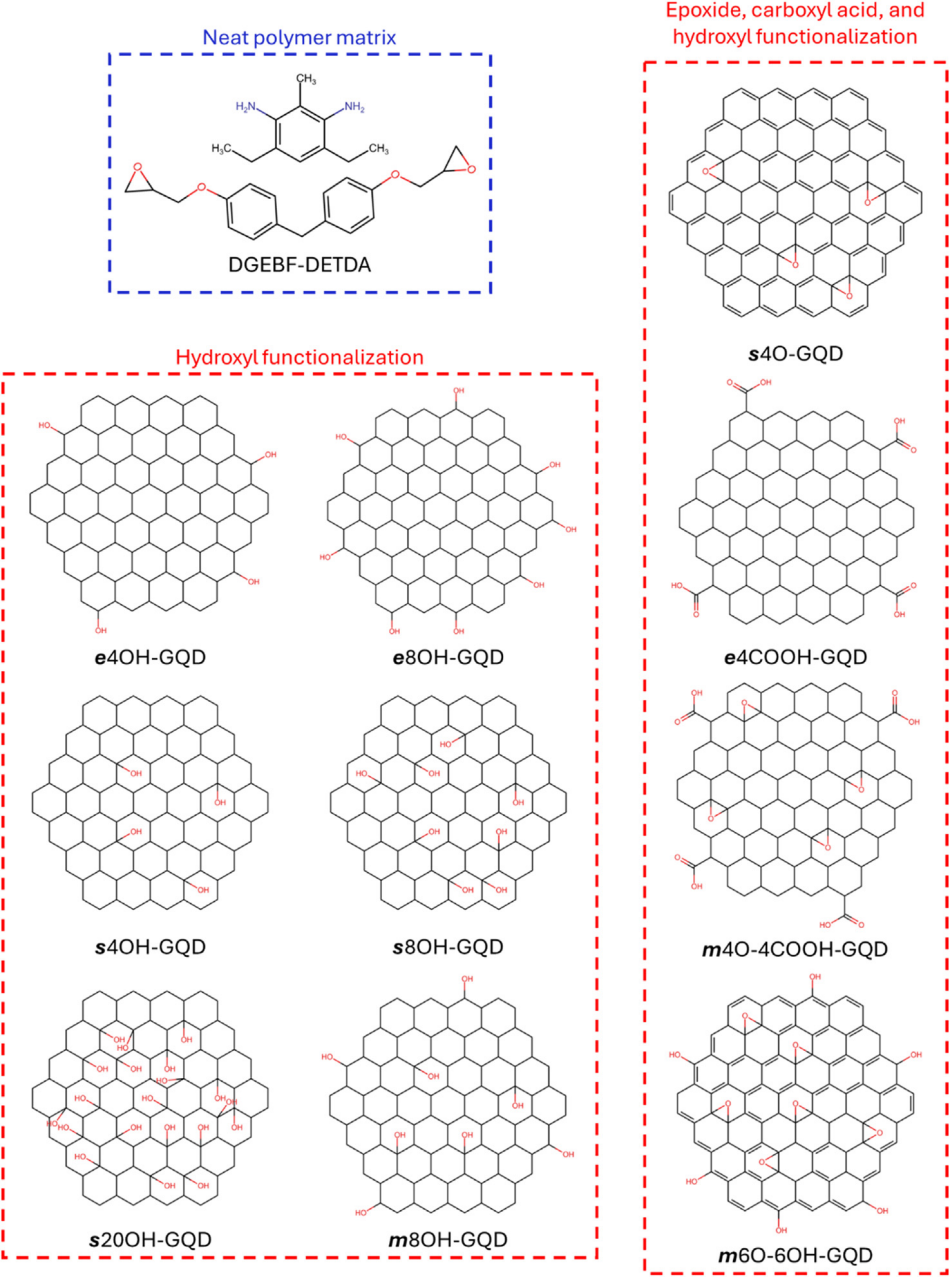
Chemical structures of all the modelled monomers.
2. The initial density of the mixed system was 0.09–0.10 g/cm^3^. Before densifying the system to the liquid density, the molecules were prepared by ramping the system temperature down from 600 K to 300 K over 100 ps (ps). A Nose-Hoover thermostat [[21], [22], [23], [24]] was used and the timestep was set to 1 fs (fs). The simulation box was gradually compressed from all directions. The compression was simulated with the rate of compression set to 10–6 Å/fs with the target density of 1.17 g/cm^3^, the liquid density of epoxy [20].
3. The densified model was annealed by raising the simulation temperature to 500 K and gradually cooling down to 400 K with a cooling rate of 50 K/ns. The annealing simulation was implemented to re-orient molecules and attain lower energy configuration. A Nose-Hoover barostat was used to maintain the simulation pressure at 1 atm. A 1 fs timestep was implemented.
4. The annealed model was maintained at 400 K and a virtual curing simulation was performed at 400 K over 2 ns using the REACTER tool within LAMMPS [25]. The simulated crosslinking reaction is the two-step amine-epoxy reaction [9,12,20]. The crosslinking simulation using REACTER requires four key inputs – frequency of new bond formation per timestep, probability of new bond formation, and minimum and maximum bond cutoff distance for formation of the new bond. For all the simulations, new bonds were formed every 10 timesteps with a 0.5 fs timestep. The probability of bond formation was maintained at 0.001 and 0.01 for the first and second step of the crosslinking reaction. The low probability values ensured low number of new bonds formed and stability of the MD model since the reaction simulation releases potential energy. High amount of energy release can result in large atomic displacements and can result in simulation failure. The bond cutoff distance was maintained between 0.5 and 6 Å.
5. The crosslinked model was re-annealed from 500 K to 300 K with a constant cooling rate of 50 K/ns. The simulation was relaxed by performing energy minimization and then cooled down to 300 K under NPT ensemble (1 atm pressure). The cooled model was then relaxed for another 0.5 ns.
6. The relaxed model was transitioned to ReaxFF forcefield [26]. The model was relaxed using the NPT ensemble at 300 K temperature and 1 atm pressure. The simulation was performed for 0.5 ns at a lowered timestep of 0.25 fs. Fig. 3 shows the MD model of the GQD-epoxy system for ***s***4OH*-*GQD.

**Fig. 3:**
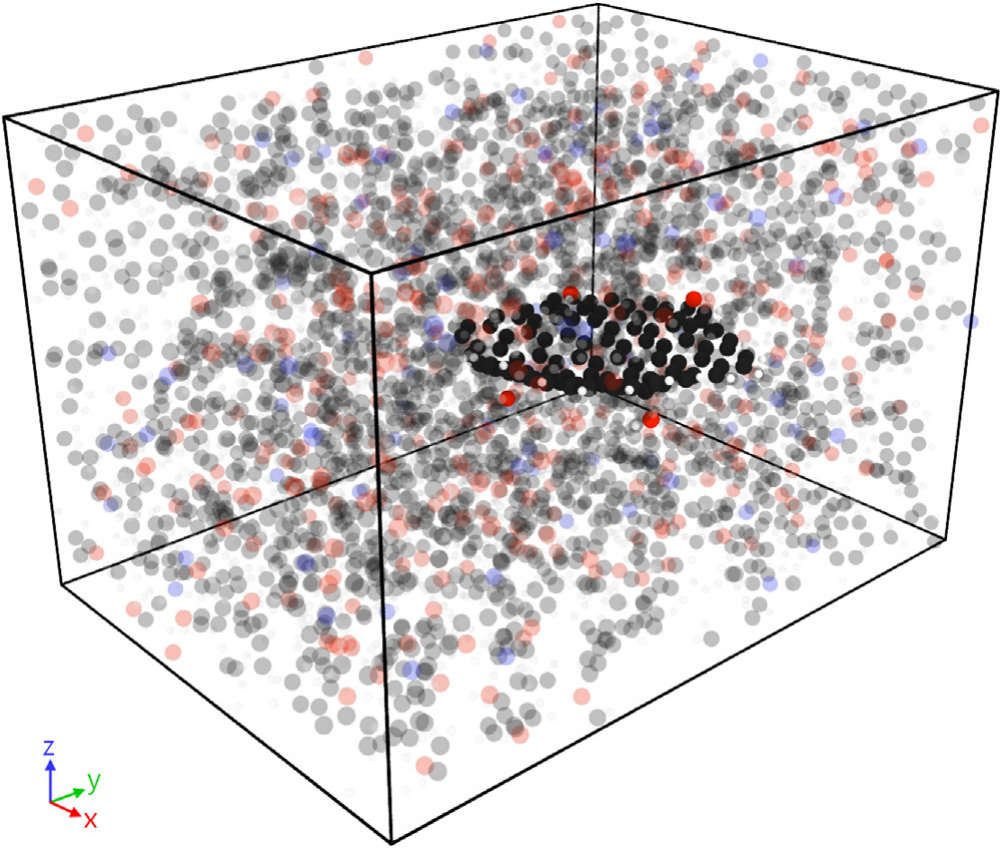
Representative MD model of epoxy (blurred) with embedded s4OH-GQD.
7. The relaxed models were then deformed in the three principal directions (*x*-, *y*-, and *z*- direction). To allow the Poisson contraction, an NPT ensemble was used to relax the lateral directions. The maximum applied strain was set at 20 % with the strain rate of 2 × 10^8^ /s and 0.1 fs timestep. The stress-strain data was then analysed to extract the Young's modulus and the yield strength of the material.

## Limitations

Not applicable

## Ethics Statement

The authors have read and follow the ethical requirements for publication in Data in Brief and confirm that the current work does not involve human subjects, animal experiments, or any data collected from social media platforms.

## Credit Author Statement

**Prathamesh P. Deshpande:** Conceptualization; Methodology; Software; Validation; Formal analysis; Investigation; Resources; Data Curation; Writing - Original Draft; Writing - Review & Editing; Visualization. **Robert Chan-Jobe:** Software; Validation; Formal analysis; Writing - Review & Editing. **Ozgur Keles:** Conceptualization; Methodology; Formal analysis; Investigation; Resources; Writing - Review & Editing; Supervision; Project administration; Funding acquisition.

## Data Availability

Mendeley DataMolecular Modeling Workflow for Optimizing Oxidized Graphene Quantum Dot and Epoxy Nanocomposite for Enhanced Mechanical Properties (Original data).
